# The American Journal and Library of Dental Science

**Published:** 1842-07

**Authors:** 


Art. III.—*The American Journal and Library of Dental Science.* Vol. II, No. 4, June, 1842. Baltimore.

There was as much truth as wit in the wag who said he never saw "M. D." after a dentist's name, that he did not read it "*miserable dentist*"; as much truth, we say, as wit, as many an unfortunate young gentleman and gentle "spinster" can abundantly testify. Of all professions or callings, of all occupations or employments, of all sciences or arts that have ever risen and flourished, we venture to give it as our firm belief that there have been more absolute, unredeemed *bunglers* and *botches*, more downright, incorrigible *ignoramuses* and *dolts*, in that of dentistry than all others put together. We have seen and heard of so much irreparable injury inflicted by them, if not with malice prepense with at least something akin to it, that we freely confess the appellation is, or rather was, any thing but a badge of knighthood, or a stamp of gentility in our view.

Such, we say, was the case; but it is a source ef pleasure to think that the day has gone by when a fellow, too lazy to work, provided with a leaf or two of gold foil, or something resembling it, an old file and a pair of forceps, could set out, advertise himself "dentist," with a long string of recommendations, and commence his murderous work of rasping, filing and filling. Men of talents, and many of them possessing high attainments, have entered the field and wrested it from the occupancy of mountebanks and greedy charlatans, driving out these unclean spirits who infested it. Both as a science and an art dentistry has made rapid advances within the last few years, and bids fair soon to attain an honorable rank among the departments of surgery. To practice this profession successfully now, not only is great mechanical skill required, but likewise an intimate acquaintance with the laws and phenommena of diseased action as it affects the whole or a part of the body. A man can set him down now in a dentist's chair (what an inviting air they all have, too!), without fear of being mangled or crucified—of undergoing tortures to which the rack, iron boots, and screws of Torquemada, or the live coals of Cortez, would be beds of down, and exquisite pleasure.

In extending the reform thus happily commenced, the Journal will prove an efficient instrument in the hands of the association by whom it is published, and afford powerful aid to the Baltimore College of Dental Surgery recently established by the same Society. For this reason, therefore, if there were none other, it commends itself to the patronage of the profession. Its appearance, to use the language of a contemporary, "is every way creditable"; indeed, as to typography, it is one of the most neatly printed journals that we have seen. No pains or expense seems to be spared by the conductors to render it valuable. The journal department of the present number, which completes the second volume, contains several interesting papers, both original and selected, and is embellished with numerous wood-cuts. The third volume commences in September next, and we hope will be issued to an increased number of subscribers. C.

